# Integrating heterogeneous knowledge graphs into drug–drug interaction extraction from the literature

**DOI:** 10.1093/bioinformatics/btac754

**Published:** 2022-11-23

**Authors:** Masaki Asada, Makoto Miwa, Yutaka Sasaki

**Affiliations:** Toyota Technological Institute, 2-12-1 Hisakata, Tempaku-ku, Nagoya, Aichi 468-8511, Japan; Toyota Technological Institute, 2-12-1 Hisakata, Tempaku-ku, Nagoya, Aichi 468-8511, Japan; Toyota Technological Institute, 2-12-1 Hisakata, Tempaku-ku, Nagoya, Aichi 468-8511, Japan

## Abstract

**Motivation:**

Most of the conventional deep neural network-based methods for drug–drug interaction (DDI) extraction consider only context information around drug mentions in the text. However, human experts use heterogeneous background knowledge about drugs to comprehend pharmaceutical papers and extract relationships between drugs. Therefore, we propose a novel method that simultaneously considers various heterogeneous information for DDI extraction from the literature.

**Results:**

We first construct drug representations by conducting the link prediction task on a heterogeneous pharmaceutical knowledge graph (KG) dataset. We then effectively combine the text information of input sentences in the corpus and the information on drugs in the heterogeneous KG (HKG) dataset. Finally, we evaluate our DDI extraction method on the DDIExtraction-2013 shared task dataset. In the experiment, integrating heterogeneous drug information significantly improves the DDI extraction performance, and we achieved an F-score of 85.40%, which results in state-of-the-art performance. We evaluated our method on the DrugProt dataset and improved the performance significantly, achieving an F-score of 77.9%. Further analysis showed that each type of node in the HKG contributes to the performance improvement of DDI extraction, indicating the importance of considering multiple pieces of information.

**Availability and implementation:**

Our code is available at https://github.com/tticoin/HKG-DDIE.git

## 1 Introduction

Drug–drug interaction (DDI) is defined as a change in the effects of one drug by the presence of another drug (Rodrigues, 2019). In order to practice ‘evidence-based medicine’ (Sackett, 1997) and prevent accidents caused by drugs, it is important to extract knowledge about DDIs from pharmaceutical papers comprehensively. Automatic DDI extraction can greatly benefit the pharmaceutical industry, providing an interesting way of reducing the time spent by healthcare professionals reviewing the medical literature.

In the research of automatic DDI extraction, the methods with convolutional neural networks (CNNs) (Liu *et al.*, 2016) and recurrent neural networks (RNNs) (Sahu and Anand, 2018) have been widely used, and these methods have shown higher performance than feature-based methods. Furthermore, especially in recent years, the BERT (Devlin *et al.*, 2019), which has adopted the Transformer (Vaswani *et al.*, 2017) architecture and pre-trained contextualized token representation on a large-scale raw text, has shown extremely high performance. Pre-training on large-scale raw texts dramatically improved the DDI extraction performance; however, existing methods consider only the context around drug mentions.

In our previous study (Asada *et al.*, 2018, 2021a), we have proposed a method of linking two drug mentions appearing in an input sentence with an entry in the drug database DrugBank (Wishart *et al.*, 2018). In Asada *et al.* (2021a), we referred to the drug description information and molecular structure information registered in DrugBank. We encoded drug description information with BERT and drug molecular structure information with graph neural networks (GNNs) (Tsubaki *et al.*, 2019), and then we combined this information and input sentence representation. We showed that using the drug description and molecular structure information, in addition to the input sentence representation of BERT, can improve the performance of DDI extraction from texts.

There is much other information related to drugs, such as protein, drug category, anatomical therapeutic chemical (ATC)-code and pathway, but no previous study confirmed the effectiveness of this heterogeneous information in DDI extraction. We are the first who represent heterogeneous drug-related knowledge, including molecular structures, in a unified vector space. We aim to integrate diverse information into the DDI extraction task, and we believe this approach provides a new way to integrate heterogeneous knowledge in relation extraction.

In this article, we propose a novel method for DDI extraction from the literature that simultaneously considers multiple drug-related information. We first obtain the heterogeneous drug representation embeddings by performing a link prediction task on the pharmaceutical knowledge graph (KG) dataset PharmaHKG (Asada *et al.*, 2021b). Then, we integrate the heterogeneous KG (HKG) representation into the input sentence representation of the DDI extraction model by the entity marker (Ye *et al.*, 2022; Zhong and Chen, 2021).

Our contributions are summarized as follows:

- We propose a novel method to effectively utilize heterogeneous pharmaceutical KG information for extracting DDIs from texts.
- We evaluate the extraction performance on DDIExtraction-2013 share task dataset (Segura-Bedmar *et al.*, 2013), and we show that heterogeneous drug information is helpful for DDI extraction and our model achieves state-of-the-art performance.

## 2 Materials and methods

This study proposes a method that utilizes multiple heterogeneous information for extracting DDIs from the literature. We first explain how we obtain the drug HKG representation, and we then describe our proposed method that combines the obtained KG embeddings and input sentence representations.

### 2.1 HKG embeddings

We obtain HKG representation embeddings of drugs by performing a link prediction task on a PharmaHKG (Asada *et al.*, 2021b) dataset. Link prediction is a task to predict an entity related to a given entity with a given relation if any. That is, typically, it is the task to predict *t* from that forms triple (*h*, *r*, *t*), given *h* and *r*. We can use the training to solve the link prediction task to represent embedding vectors for the nodes and the links in a KG. For KGs are always imperfect, link prediction aims to discover and add missing knowledge into it. With the existing relations and entity, candidate entities are selected to form a new fact. We replace the head or tail of the triples in the validation or test dataset with other entities that have the same entity types and calculate the scores of all created negative triples in the KG. We sort the calculated positive triple score and the scores of all negative triples and evaluate the rank of the positive triple score.

The PharmaHKG dataset consists of the following five types of nodes:

- **Drug**: We extract information on drugs from DrugBank (Wishart *et al.*, 2018). More than 10 000 drugs are registered in DrugBank, and various types of information, such as drug names, descriptions, molecular structures and experimental properties, are registered.
- **Protein**: We extract the protein information from UniProt (UniProt Consortium, 2018). UniProt consists of Swiss-Prot, manually annotated and reviewed, and TrEMBL, automatically annotated and not reviewed, and we use the Swiss-Prot knowledgebase.
- **Pathway**: We extract information on pathways from Small Molecule Pathway Database (SMPDB) (Jewison *et al.*, 2014). SMPDB is an interactive, visual database containing more than 30 000 small molecule pathways found in humans.
- **Category**: We extract information on drug categories from the medical thesaurus Medical Subject Headings (MeSH) (Lipscomb, 2000). Each drug recorded in DrugBank has several hypernymy categorical classes, and these classes have the corresponding MeSH term IDs.
- **ATC**: ATC classification system also has categorical information on drugs. In the ATC classification system, drugs are divided into different groups according to the organ or system on which they act and their therapeutic, pharmacological and chemical properties. The ATC classification system classifies drugs into groups at five different levels.

The following eight types of relations connect these nodes:

- *category*: Drug nodes and MeSH nods are linked by this relation.
- *ATC*: Drug nodes and ATC classification system code nodes are linked by this relation.
- *pathway*: Drug/protein nodes and pathway nodes are linked by this relation.
- *interact*: Drug nodes are linked by this relation when concomitant use of the pair of drugs will affect its activity or result in adverse effects. This link does not distinguish between synergistic and antagonistic interactions.
- *target*: Drug nodes and protein (a protein that binds to a given drug, resulting in an alteration of the normal function) nodes are linked by this relation.
- *enzyme*: Drug nodes and protein (a protein that catalyzes chemical reactions involving a given drug) nodes are linked by this relation.
- *carrier*: Drug nodes and protein (a secreted protein that binds to drugs, carrying them to cell transporters) nodes are linked by this relation.
- *transporter*: Drug nodes and protein (a membrane-bound protein that shuttles ions into cells or out of cells) nodes are linked by this relation.

Logistic loss is commonly used for KG embedding training. The logistic loss returns −1 for negative samples and +1 for the positive samples. Negative samples are created by corrupting triples (*h*, *r*, *t*). The model corrupts *h* or *t* by sampling from a set of head or tail entities for heads and tails, respectively. The corrupted triples can be either of (h′,r,t) or (h,r,t′), where h′ and t′ are replaced head and tail entities. $D^{+}$ and $D^{-}$ are negative and positive data, y=±1 is the label for positive and negative triples, and f(·) is the score function. Model parameters are trained by minimizing the negative log-likelihood of the logistic model with *L*2 regularization on the parameters Θ of the model;

(1) $$L_{KG}=\sum_{(h,r,t)\in D^{+}\cup D^{-}}\;\text{log}\;(1+\text{exp}(y\times f(h,r,t)))+\lambda ||\Theta |{|}_{2}^{2}.$$

Score function f(h,r,t) is defined on each triple (*h*, *r*, *t*) to access the validity of triples. Triples observed in the KG tend to have higher scores than those that have not been observed. We employ the following four score functions: TransE (Bordes *et al.*, 2013), DistMult (Yang *et al.*, 2014), ComplEx (Trouillon *et al.*, 2016) and SimplE (Kazemi and Poole, 2018).

**Fig. 1.**
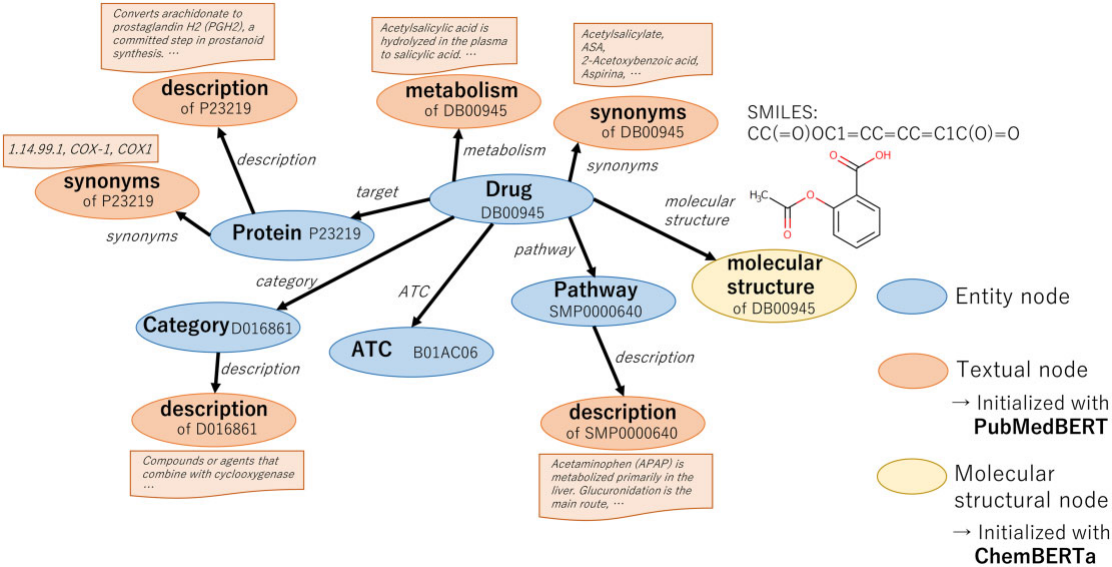
Construction of heterogeneous pharmaceutical KG

We make one extension from the HKG by Asada *et al.* (2021b); i.e. the molecular structural nodes of the drugs are added to the HKG. An overview of the newly constructed KG is shown in Figure 1. Similar to initializing textual nodes with embeddings by a pre-trained BERT model, we also initialize molecular structural nodes with a pre-trained model of the SMILES string coding representation embeddings (Chithrananda *et al.*, 2020).

The constructed pharmaceutical HKG enables us to obtain drug representation vectors that take into account various information such as hierarchical categorical information, interacting protein information, related pathway information, textual drug information and drug molecular structural information.

### 2.2 DDI extraction using KG embeddings

DDI extraction is a task to identify drug pairs in an input sentence in which the interaction of the pairs is described and assign the right types of interactions to the pairs. The task of extracting DDIs consists of two parts: named entity recognition and relation extraction (RE). In this study, we focus on the RE part, assuming drug entities are given, following existing methods (e.g. Liu *et al.*, 2016). We treat the extraction of DDIs from text as a multi-class classification problem, where a part of the target drug mentions and the remaining drug mentions are specified in the input sentence.

**Fig. 2.**
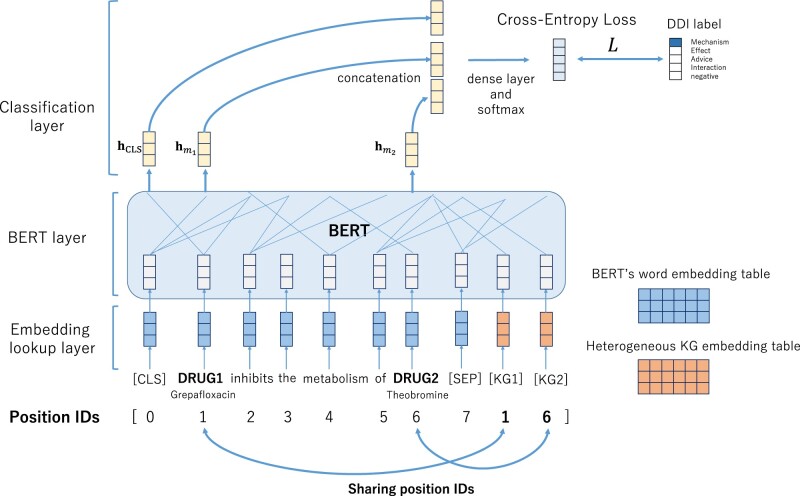
Overview of our DDI extraction model

Figure 2 shows an overview of our novel DDI extraction model from the literature using the HKG representations of drugs obtained in the previous section. Our proposed model adopts the idea of an entity marker (Ye *et al.*, 2022; Zhong and Chen, 2021), and we place two drug mention markers at the end of a sentence.

*Embedding layer*. The input sentence *S* is tokenized into sub-word units by the BERT tokenizer and converted to the format shown below:

(2) $$S=\{[\mathrm{CLS}],w_{1},\ldots ,w_{m_{1}},\ldots ,w_{m_{2}},\ldots ,[\mathrm{SEP}],[\mathrm{KG1}],[\mathrm{KG2}]\},$$

where *w_i_* is the *i*th sub-word, [CLS] and [SEP] are the special tokens of BERT, *m*_1_ is the drug mention 1 (*DRUG1*), *m*_2_ is the drug mention 2 (*DRUG2*), and [KG1] and [KG2] are markers for mapping mentions and KG entries.

Then, in the embedding lookup layer of the BERT model, the sub-word *w_i_* and special tokens are converted to embedding vectors by the pre-trained BERT embedding table. In addition, we initialize the marker embeddings with the HKG embeddings. All tokens are converted to embedding vectors, and the embedding matrix $W^{0}$ of the input sentence is shown as follows:

(3) $$W^{0}=\{w_{\mathrm{CLS}},w_{1},\ldots ,w_{m_{1}},\ldots ,w_{m_{2}},\ldots ,w_{\mathrm{SEP}},w_{\mathrm{KG1}},w_{\mathrm{KG2}}\},$$

where $w_{i},\;w_{\mathrm{CLS}}$ and $w_{\mathrm{SEP}}$ are from the BERT embedding table $V_{\mathrm{BERT}}\in R^{N_{v}\times d}$ and $w_{\mathrm{KG1}}$, while $w_{\mathrm{KG2}}$ are from the HKG embedding table $V_{KG}\in R^{N_{e}\times d}$. Here, *d* is the dimension of the embedding vector, *N_v_* is the number of vocabularies of the BERT tokenizer, and *N_e_* is the number of entities in the HKG.

*Self-attention layer.* The embedding matrix $W^{0}$ is the input to the *L*-layers of the BERT self-attention module:

(4) $$W^{l+1}={\text{SelfAttention}}^{l}(W^{l}),$$

where l=0,1,2,…,L−1. The output of the final attention layer $W^{L}$ is shown as follows:

(5) $$W^{L}=\{h_{\mathrm{CLS}},h_{1},\ldots ,h_{m_{1}},\ldots ,h_{m_{2}},\ldots ,h_{\mathrm{SEP}},h_{\mathrm{KG1}},h_{\mathrm{KG2}}\},$$

where $h_{i}\in R^{d}$ is the hidden state vector of *i*th token. As shown in Figure 2, each mention and its KG entity share the position ID, which maps the mention and the marker.

*Prediction layer.* We calculate the loss function from the hidden representation vectors of the final layer of the BERT architecture. First, we concatenate the hidden representation of the CLS token and two drug-mention tokens as follows:

(6) $$h_{\mathrm{all}}=[h_{\mathrm{CLS}};h_{m_{1}};h_{m_{2}}].$$

The concatenated representation vector $h_{\mathrm{all}}$ is passed through a dense layer, and we obtain the middle layer representation,

(7) $$h_{\mathrm{mid}}=W_{\mathrm{mid}}^{}h_{\mathrm{all}}+b_{\mathrm{mid}}^{},$$

where $W_{\mathrm{mid}}\in R^{3d\times d_{m}},\;b_{\mathrm{mid}}\in R^{d_{m}}$ are the trainable weights and biases, and *d_m_* is the dimension of middle layer vector. Then, the middle layer representation is converted into a fully connected representation as follows:

(8) $$h_{fc}=W_{fc}^{}h_{\mathrm{mid}}+b_{fc}^{},$$

where $W_{fc}\in R^{d_{m}\times c},\;b_{fc}\in R^{c}$ are the trainable weights and biases, and *c* is the number of label types. The fully connected representation vector $h_{fc}$ is converted to probability form by the softmax function. The model parameters are updated to minimize the cross-entropy loss.

## 3 Experiments

### 3.1 Preprocessing of input sentences

We follow Liu *et al.* (2016) to preprocess the input sentences. When three or more drug mentions appear in an input sentence, we duplicate the sentence for each drug mention pair. Specifically, if an input sentence contains *n* drug mentions, $\left(\begin{array}{c} n\\ 2 \end{array}\right)$ input sentences with different drug mention pairs are prepared. We preprocess each input sentence to specify the target drug mention pair and other drugs. In detail, we replace the target drug pair with DRUG1 and DRUG2 in the sentence order and replace other drugs with DRUGOTHER. We show the example of the preprocessing on the sentence *Exposure to oral S-ketamine is unaffected by itraconazole but greatly increased by ticlopidine* with different target drug pairs in Table 1.

**Table 1.** An example of preprocessing

Mention1	Mention2	Preprocessed input sentence
*S-ketamine*	*itraconazole*	*Exposure to oral* ***DRUG1****is unaffected by* ***DRUG2****but greatly increased by DRUGOTHER.*
*S-ketamine*	*ticlopidine*	*Exposure to oral* ***DRUG1****is unaffected by DRUGOTHER but greatly increased by* ***DRUG2***.
*itraconazole*	*ticlopidine*	*Exposure to oral DRUGOTHER is unaffected by* ***DRUG1****but greatly increased by* ***DRUG2***.

*Note*: The input sentence ‘Exposure to oral S-ketamine is unaffected by itraconazole but greatly increased by ticlopidine’ contains three target drug pairs.

### 3.2 Drug mention linking

We link drug mentions not only with DrugBank drug entities but also with MeSH categorical terms, ATC code categorical terms and UniProt protein terms. The DDIExtraction-2013 shared task dataset consists of four types of entities, *DRUG*, *DRUG_N*, *BRAND* and *GROUP*. The *GROUP* type drug mentions may be linked to categorical terms.

As a result, 97.05% of unique mentions in the training dataset were linked to HKG entries, and 97.89% of unique mentions in the test dataset were linked. As for the coverage on relation instances where both drug mentions are linked, the coverage of the train data instances was 91.90% (25 540/27 792), and the coverage of the test instances was 90.75% (5187/5716).

### 3.3 Link prediction task settings

We used the same train/validation/test split triples as the datasets created by Asada *et al.* (2021b). The statistics of the number of nodes is shown in Table 2, and the statistics of the KG edges for each relation type is shown in Table 3. We employed mini-batch training using the Adagrad (Duchi *et al.*, 2011) optimizer. We performed hyperparameter tuning on the validation dataset. Hyper-parameters include an initial learning rate and a mini-batch size.

**Table 2.** Statistics of heterogeneous pharmaceutical KG entities

Entity type	*N*
Drug (DrugBank-ID)	11 516
Protein (Uniprot-ID)	5339
Pathway (SMPDB-ID)	874
Category (MESH-ID)	2166
ATC (ATC-code)	1093

Total	20 988

**Table 3.** Statistics of heterogeneous pharmaceutical KG edges for each relation type

Relation type	ALL	Train	Valid	Test
Category	60 459	54 419	3020	3020
ATC	16 341	14 711	815	815
Pathway	18 707	16 847	930	930
Interact	2 682 142	2 413 932	134 105	134 105
Target	18 467	16 627	920	920
Enzyme	5206	4686	260	260
Carrier	815	735	40	40
Transporter	3093	2793	150	150

Total	2 750 228	2 525 829	140 240	140 240

We extracted SMILES strings from the DrugBank database. The 9859 drug entities in the HKG dataset have SMILES strings. Relation triples (drug, *structure* and SMILES) are added to the train dataset, and molecular structural nodes (SMILES nodes) are initialized by the embedding vectors of pre-trained SMILES representation language model ChemBERTa (Chithrananda *et al.*, 2020).

We used CLS token representation of PubMedBERT (Gu *et al.*, 2021) as the initial value of the textual nodes and ChemBERTa as the initial value for the molecular structural nodes. ChemBERTa is the model that was pre-trained on 77M unique SMILES from PubChem (Kim *et al.*, 2021), the world’s largest open-source collection of chemical structures. The SMILES were canonicalized and globally shuffled to facilitate large-scale pre-training. ChemBERTa is based on the RoBERTa (Liu *et al.*, 2019) model. In pre-training, the ChemBERTa model masks 15% of the tokens in each SMILES string.

### 3.4 DDI extraction task settings

We followed the DDIExtraction-2013 (Segura-Bedmar *et al.*, 2013) shared task settings. This dataset is composed of input sentences containing the drug mention pair, and the following four DDI types are annotated to each drug pair.

- *Mechanism*: this type is assigned when a pharmacokinetic interaction is described in an input sentence.
- *Effect*: this type is assigned when a pharmacodynamic interaction is described in an input sentence.
- *Advice* this type is assigned when a recommendation or advice regarding the concomitant use of two drugs is described in an input sentence.
- *Interaction* (*Int.*): this type is assigned when the sentence states that interaction occurs and does not provide any detailed information about the interaction.

Table 4 shows the statistics of DDI extraction dataset. We performed 5-fold cross-validation on the train dataset while keeping the distribution of labels in each split. We used the results for hyper-parameter tuning, model architecture selection and error analysis. After tuning the model, we evaluated the performance on the test set to compare it with existing methods.

**Table 4.** Statistics of SemEval-2013 DDIExtraction task dataset

	Train	Test
Documents	714	191
Sentences	6976	1299
Drug pairs	27 792	5716
Positive drug pairs	4021	979
Mechanism	1319	302
Effect	1687	360
Advice	826	221
Int.	189	96
Negative drug pairs	23 771	4737

We employed the AdamW optimizer (Loshchilov and Hutter, 2019), and we employed mixed-precision training (Le Gallo *et al.*, 2018) for memory efficiency. We employed the weight averaging (Polyak and Juditsky, 1992) technique, where all model parameters are saved at each update, and the model predicts the DDI label from the average of all stored parameters. We employed PubMedBERT as the textual representation model for the DDI extraction task. The word embeddings of PubMedBERT and HKG embeddings are frozen during training. The hyper-parameters include a learning rate, a weight decay coefficient, a dropout probability and a mini-batch size. Our significance tests are based on the Randomized Shuffle test (Fisher *et al.*, 1937). We set the number of shuffles to 10 000.

### 3.5 Additional case study: the DrugProt task

In order to verify the generality of our proposed model, we evaluated our model on a dataset other than the DDI corpus. We used the dataset from the BioCreative VII Track 1 - Text mining drug and chemical–protein interactions (DrugProt) (Miranda *et al.*, 2021) for the evaluation. The DrugProt dataset is composed of documents manually annotated with drug mentions, protein mentions and their relations. The DrugProt corpus consists of training, development and test sets. Since the gold-standard annotations for the test set are not publicly available at present, we conducted hyper-parameter tuning on the training set and evaluated the model on the development set. The DrugProt dataset contains 17 288 relations for the training set and 3765 relations for the development set. We followed the settings of the DrugProt task. The task is to classify a given pair of a drug and a protein into 13 relation types or no relation. We used the official evaluation script (https://github.com/tonifuc3m/drugprot-evaluation-library) provided by the task organizers. As with the DDIExtraction-2013 dataset, a randomized shuffle test with a random count of 10 000 is employed for the significance test.

## 4 Results and discussions

### 4.1 Link prediction results

We show the results of the link prediction task on the HKG in Table 5. For each of the four score functions, TransE, DistMult, ComplEx and SimplE, we evaluate the four methods listed below:

**Table 5.** The comparison of link prediction performance on heterogeneous KG

	TransE	DistMult	ComplEx	SimplE
Entity nodes only				
MRR	**0.3114**	0.6732	0.6627	0.6228
Hits@1	**0.0108**	0.5416	0.5436	0.3954
Hits@3	**0.5590**	0.7680	0.7377	0.8287
Hits@10	**0.7364**	0.9138	0.8892	0.9481
With textual nodes				
MRR	0.2894	0.7702	0.7874	0.7175
Hits@1	0.0092	0.6199	0.6424	0.5019
Hits@3	0.5102	0.9104	0.9258	0.9303
Hits@10	0.6987	0.9703	0.9722	0.9744
With molecular structural nodes				
MRR	0.3003	0.7677	0.7313	0.7156
Hits@1	0.0094	0.6171	0.5383	0.4987
Hits@3	0.5352	0.9092	0.9180	0.9307
Hits@10	0.7195	0.9700	0.9717	0.9746
With textual nodes and molecular structural nodes				
MRR	0.2877	**0.7933**	**0.7923**	**0.7235**
Hits@1	0.0091	**0.6610**	**0.6509**	**0.5086**
Hits@3	0.5051	**0.9166**	**0.9279**	**0.9386**
Hits@10	0.6995	**0.9711**	**0.9729**	**0.9753**

*Note*: The comparison of link prediction performance on heterogeneous KG. Figures marked in bold indicate the highest performance when limited to each score function. The underlines indicate the highest performance among all score functions.

- **entity nodes only**: This is a method that trains the link prediction model only from the nodes in the HKG. In Figure 1, only the actual nodes (the blue ones) are included in the train dataset.
- **with textual nodes**: In this method, in addition to the actual nodes, pseudo nodes that hold textual information, such as synonyms and descriptions of entity items, are added to the HKG. In Figure 1, textual nodes (the red one) are added to the actual nodes.
- **with molecular structural nodes**: In this method, pseudo molecular structural nodes are added to the HKG in addition to the actual nodes. As shown in Figure 1, molecular structural nodes (the yellow ones) are added.
- **with textual nodes and molecular structural nodes**: In this method, both textual nodes and molecular structural nodes are added. This approach can consider a wide variety of heterogeneous information about drugs.

Table 5 showed that the TransE model performs poorly for both MRR and Hits@*k*, which should be due to the inability of the TransE model to capture the symmetrical relational triples. The TransE model showed low performance of MRR and Hits@*k* because our HKG contained a large proportion of the (drug, *interact* and drug) triples, which is a symmetric relationship. Furthermore, the TransE model showed the highest MRR and Hits@*k* when using the ‘entity nodes only’ method, meaning that adding textual or molecular structural nodes did not improve link prediction performance.

On the other hand, DistMult, ComplEx and SimplE, which can consider symmetric relationships, showed higher performance than TransE. These models successfully improved the performance of the link prediction task by adding textual nodes and molecular structural nodes, respectively. Further performance improvement was achieved when both textual and molecular structural nodes were added. As shown by the underlined scores in Table 5, the highest performance for all MRR and Hits@*k* metrics was achieved by the method using both textual and molecular structural nodes. These results show that rich embedding representations are obtained by considering various heterogeneous domain information.

### 4.2 DDI extraction results

We describe the performance of DDI extraction models that leverage these HKG embeddings. Table 6 shows the performance evaluated on the DDIExtraction-2013 task test set. Our proposed model PubMedBERT+HKG achieved an F-score of 85.40%, showing the current state-of-the-art performance. In addition, the proposed model achieved a significant F-score improvement of 1.70 percent points (pp) over the baseline model by using heterogeneous information about drugs. Compared to other existing models, our PubMedBERT+HKG model showed a higher F-score. The SciBERT+Mol.+Desc. model is an ensemble of SciBERT with the drug molecular structure information and SciBERT with the drug description information. Our proposed model showed higher performance than the ensemble of multiple models.

**Table 6.** The comparison of DDI extraction performance on the SemEval-2013 test dataset

Method	P	R	F (%)
Reported scores			
CNN (Liu *et al.*, 2016)	75.29	60.37	67.01
BiLSTM (Sahu and Anand, 2018)	67.77	66.80	67.28
PubMedBERT (Gu *et al.*, 2021)	–	–	82.42
SciFive-Large (Phan *et al.*, 2021)	–	–	83.67
Our implementation			
SciBERT + Mol. + Desc. (Asada *et al.*, 2021a)	85.36	82.83	84.08
PubMedBERT (baseline)	83.45	83.96	83.70
**PubMedBERT + HKG**	85.32	85.49	**85.40** ^a^

HKG, heterogeneous knowledge graph.

The highest F-scores are shown in bold.

a Indicates performance improvement from PubMedBERT (baseline) at a significance level of *p *<* *0.005.

Then, we show the F-score for each of the four DDI labels of DDIExtraction-2013 task dataset in Table 7. As shown in Table 7, the model with HKG information improved the F-score from the baseline model by 2.42 pp for the *Mechanism* label, 1.52 pp for the *Effect* label and 1.67 pp for the *Advise* label. For the *Int.* label, the proposed model showed a slightly lower F-score than the baseline model.

**Table 7.** The comparison of F-scores for individual DDI types on the SemEval-2013 test dataset

Methods		Mech.	Effect	Adv.	Int.
PubMedBERT (baseline)	P	87.12	87.18	78.71	82.69
	R	85.10	92.31	88.33	44.79
	F	86.10	89.67	83.25	**58.11**
PubMedBERT + HKG	P	88.96	88.84	81.06	81.13
	R	88.08	93.67	89.17	44.79
	F	**88.52**	**91.19**	**84.92**	57.72

The highest F-scores are shown in bold.

#### 4.2.1 Selecting score functions

This section will discuss which score function was effective for DDI extraction. Table 8 shows the average F-scores for the five validation datasets for each score function. F-score is higher than the baseline model when using HKG embeddings trained by any score functions. The improvement of F-score points from the baseline model is 1.28 for the TransE model and 1.94 for the DistMult model and 1.36 for the ComplEx model and 1.32 for the SimplE model. From these results, we adopted the DistMult score function for our DDI extraction model. The DistMult model performed best on the link prediction task on MRR, Hits@1 and also showed the best performance on the DDI extraction task.

**Table 8.** Comparison of DDI extraction performances with different score functions for training KG embeddings

Methods	P	R	F (%)
PubMedBERT (baseline)	82.19	83.23	82.54
+ HKG (TransE)	83.46	84.37	83.82
+ HKG (DistMult)	83.68	85.42	**84.48**
+ HKG (ComplEx)	83.68	84.32	83.90
+ HKG (SimplE)	83.46	84.50	83.86

*Note*: We show the performance with 5-fold cross-validation on the training dataset.

The highest F-scores are shown in bold.

#### 4.2.2 Ablation study on model architecture

In this section, we provide ablation studies. Table 9 shows the ablation study results.

**Table 9.** The ablation study on the model architecture

Methods	P	R	F (%)	Δ (pp)
Five-fold cross-validation				
PubMedBERT + HKG	83.68	85.42	**84.48**	–
w/o sharing position ids	82.49	86.04	84.18	0.30
w/o freezing KG embeddings	83.69	84.32	83.90	0.58
w/o CLS representation	82.63	85.17	83.81	0.67
w/o mention representation	82.07	85.69	83.78	0.70
PubMedBERT (baseline)	82.19	83.23	82.54	1.94
Test set				
PubMedBERT + HKG	85.32	85.49	**85.40**	–
w/o sharing position ids	83.77	84.37	84.07	1.33
w/o freezing KG embeddings	85.59	84.98	85.28	0.12
w/o CLS representation	83.74	83.14	83.44	1.96
w/o mention representation	82.65	84.67	83.65	1.75
PubMedBERT (baseline)	83.45	83.96	83.70	1.70

*Note*: We showed the performance with 5-fold cross-validation on the training set and the performance on the test set.

The highest F-scores are shown in bold.

*w/o sharing position IDs*. First, we discuss the case, excluding the sharing of position IDs. The sharing of position IDs has the effect of linking the mention embeddings and KG embeddings. As shown in the Figure 2, with position sharing, the position ID of KG1 is 1 of the drug mention 1 and the position ID of KG2 is 6 of the drug mention 2. When this sharing is disabled, the position IDs of KG1 and KG2 are the values following from the IDs of the SEP token. From Table 9, the F-score is reduced from the full model when position sharing is excluded.

*w/o freezing KG embeddings*. In our proposed model, the BERT embeddings and the KG embeddings are frozen, and the attention weights are trainable. Table 9 shows that when embedding freezing is disabled, F-score is lower than the full model. We think embedding freezing is effective because if the embedding is not frozen, there will be a gap between the KG embeddings of drugs that appear on the train set and those that appear only on the test set.

*w/o CLS representation*. When $h_{\mathrm{CLS}}$ was excluded from the input vector of the middle layer, the F-score decreased. The CLS token representation holds information of the entire sentence. Therefore, it is effective to use the representation of CLS token.

*w/o mention representation*. When $h_{m_{1}}$ and $h_{m_{2}}$ were excluded from the input vector of the middle layer, the F-score decreased. In addition to the CLS token representation, it is effective to use the drug-mention representation.

*w/o KG embeddings*. Finally, we discuss the case without KG embeddings. In the baseline model, KG1 and KG2 tokens are not fed to the BERT architecture, and KG embeddings are not used. Except for this point, the model structure is the same as the proposed model. Using HKG information improved the F-score by 1.94 pp on the cross-validation and 1.70 pp on the test set. This result shows that the heterogeneous drug information positively impacts the DDI extraction from the literature.

#### 4.2.3 Ablation study on HKG node types

Table 10 shows the ablation study on the effect of individual KG node type. As shown in Table 10, all types of nodes in the HKG contribute to the performance improvement of DDI extraction from the literature. The results show the importance of simultaneously considering multiple pieces of drug-related information. Among the types of nodes, the MeSH categorical information contributed the most to the performance improvement, while the pathway information contributed the least.

**Table 10.** The ablation study on entity items

Method	P	R	F (%)	Δ (pp)
5-fold cross-validation				
PubMedBERT + HKG	83.68	85.42	**84.48**	–
w/o Protein nodes	83.22	85.04	84.08	0.40
w/o MeSH category nodes	83.28	83.75	83.38	1.10
w/o ATC nodes	83.33	84.27	83.73	0.75
w/o Pathway nodes	83.98	84.85	84.30	0.18
w/o Textual nodes	83.75	84.20	83.86	0.62
w/o Molecular structure nodes	83.36	84.30	83.71	0.77
Test set				
PubMedBERT + HKG	85.32	85.49	**85.40**	–
w/o Protein nodes	83.41	84.26	83.84	1.56
w/o MeSH category nodes	84.79	83.75	84.27	1.13
w/o ATC nodes	84.42	83.04	83.72	1.68
w/o Pathway nodes	84.39	83.96	84.17	1.23
w/o Textual nodes	84.01	83.75	83.88	1.52
w/o Molecular structure nodes	84.64	84.47	84.56	0.84

*Note*: We showed the performance with 5-fold cross-validation on the training set and the performance on the test set.

The highest F-scores are shown in bold.

#### 4.2.4 Analysis of prediction results

**Fig. 3.**
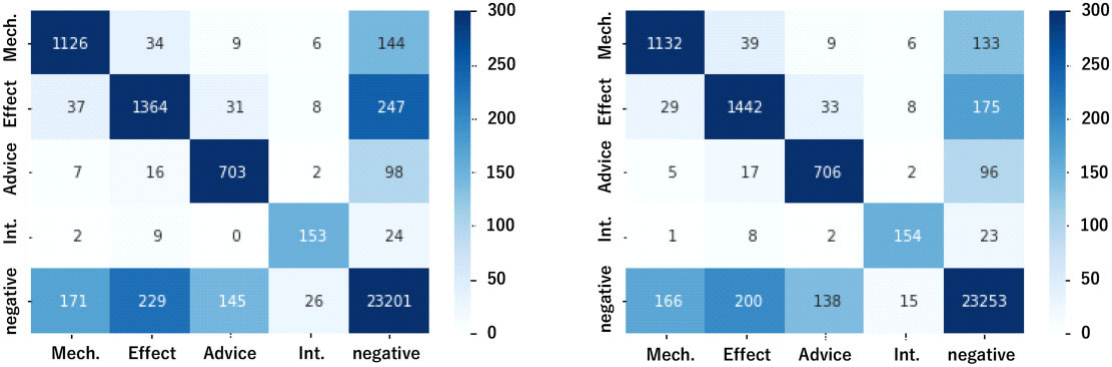
The confusion matrices [left: PubMedBERT (baseline), right: proposed method]. The vertical axis shows the actual labels, and the horizontal axis shows the predicted labels. The numbers indicate the total count of five validation datasets

The confusion matrices of the baseline and the proposed model are shown in Figure 3. The numbers indicate the total count of five validation datasets. The proposed method reduced all the patterns of errors (non-diagonal components in the table) compared to the baseline model. In particular, errors in which the model incorrectly predicts the *Effect* interaction as negative and errors in which the model incorrectly predicts the negative as *Effect* are greatly reduced. For *Mechanism*, *Advice* and *Int.* relations, the use of HKG information also reduced the number of cases of false negative or false positive relations. On the other hand, there were cases in which the use of HKG information slightly increased errors by classifying relations into wrong types, e.g. incorrectly classifying a *Mechanism* relation as *Effect*.

In addition, we show four examples of prediction results. Examples 1, 2 and 3 in Table 11 are cases correctly predicted using HKG information but incorrectly predicted by the baseline model. Many drug entities appear in Examples 1 and 2, and DRUG1 and DRUG2 are included in parentheses. As shown in Example 3, the baseline model predicts cases where the distance between DRUG1 and DRUG2 is extremely short as negative, but the proposed model correctly predicts them. From these examples, HKG representation of the drug entities may be helpful to predict correct relations when the prediction is difficult only from their surrounding contexts in the sentences.

**Table 11.** Case studies of our proposed model

Example 1
**Text**: *In patients receiving nonselective DRUGOTHER (DRUGOTHER) (e.g.* ***DRUG1****) in combination with DRUGOTHER (e.g. DRUGOTHER, DRUGOTHER, DRUGOTHER, DRUGOTHER*, ***DRUG2****), there have been reports of serious, sometimes fatal, reactions.*
DRUG1: *selegiline hydrochloride*, DRUG2: *venlafaxine*
**Gold label**: Effect **Baseline**: negative **Ours**: Effect
Example 2
**Text**: *DRUGOTHER: In a study of 7 healthy male volunteers*, ***DRUG1****treatment potentiated the blood glucose lowering effect of DRUGOTHER (a DRUGOTHER similar to* ***DRUG2****) in 3 of the 7 subjects.*
DRUG1: *acitretin*, DRUG2: *chlorpropamide*
**Gold label**: negative **Baseline**: Effect **Ours**: negative
Example 3
**Text**: *Caution should be exercised when considering the use of* ***DRUG1****and* ***DRUG2****in patients with depressed myocardial function.*
DRUG1: *BREVIBLOC*, DRUG2: *verapamil*
**Gold label**: Advice **Baseline**: negative **Ours**: Advice
Example 4
**Text**: *To determine whether* ***DRUG1****has a direct effect on the distribution of* ***DRUG2****, the elimination and distribution of DRUGOTHER was studied in six patients, five lacking kidney function and one with a partially impaired renal function, in the presence or absence of DRUGOTHER.*
DRUG1: *probenecid*, DRUG2: *cloxacillin*
**Gold label**: negative **Baseline**: negative **Ours**: Mechanism

Example 4 is a case incorrectly predicted by using HKG information while correctly predicted by the baseline model. According to the annotation guideline of the DDIExtraction-2013 dataset, an interaction should only be annotated when it occurs in the text. Example 4 shows some studies given interactions were performed; however, the sentence does not provide any evidence. In such a case, background knowledge of drugs may have disturbed correct prediction.

### 4.3 DrugProt results

The performance evaluation of drug–protein interaction extraction on the DrugProt development dataset is shown in Table 12. The result shows that using heterogeneous information about drug mentions and protein mentions achieved a significant F-score improvement of 1.4 pp. Our method showed competitive performance compared to other existing methods that use large pre-trained encoders and/or distant supervision data. These results show that the proposed method is effective for datasets other than DDI extraction. If we employ an ensemble method for the DrugProt task, e.g. Weber *et al.* (2021) and Luo *et al.* (2021), we can expect better performance; however, it is beyond the scope of this article.

**Table 12.** The comparison of drug-protein interaction extraction performance on the DrugProt dataset

Method	P	R	F (%)
Reported scores			
BioRoBERTa-Large (Yoon *et al.*, 2021)	–	–	77.4
BioRoBERTa-Large + DA (Yoon *et al.*, 2021)	–	–	78.6
PubMedBERT + DS (Iinuma *et al.*, 2022)	–	–	77.0
BioRoBERTa-Large + DS (Iinuma *et al.*, 2022)	–	–	78.0
Our implementations			
PubMedBERT (baseline)	77.2	75.9	76.5
**PubMedBERT + HKG**	78.0	77.8	77.9^a^

*Note*: We only show the single model performance for a fair comparison.

DA, data augmentation; DS, distant supervision.

a Indicates performance improvement from PubMedBERT (baseline) at a significance level of *p *<* *0.005.

Table 13 shows a comparison between the baseline model and our proposed model on F-scores for each relation type. The F-scores for INDIRECT-DOWNREGULATOR, ACTIVATOR, PRODUCT-OF and PART-OF have been especially improved. For INDIRECT-UPREGULATOR, AGONIST and SUBSTRATE relations, the proposed model showed lower F-scores than the baseline model, but the decrease in the F-score was relatively small.

**Table 13.** The comparison of F-scores for each individual drug–protein interaction type

Relation type	PubMedBERT	PubMedBERT + HKG
INDIRECT-DOWNREGULATOR	75.5	77.9
INDIRECT-UPREGULATOR	76.3	75.2
DIRECT-REGULATOR	64.2	67.5
ACTIVATOR	75.7	77.1
INHIBITOR	84.5	85.3
AGONIST	79.8	79.0
AGONIST-ACTIVATOR	0.0	0.0
AGONIST-INHIBITOR	0.0	0.0
ANTAGONIST	91.3	91.9
PRODUCT-OF	59.2	66.9
SUBSTRATE	71.0	70.8
SUBSTRATE_PRODUCT-OF	0.0	0.0
PART-OF	70.5	75.5

## 5 Conclusion

In this article, we propose a novel method for DDI extraction from the literature that integrates heterogeneous pharmaceutical KG information. We first added the drug molecular structure information to the PharmaHKG dataset and performed the link prediction task. The results showed that the MRR and Hits@*k* were improved by considering the molecular structure information of drugs, and we obtained the heterogeneous drug representation from the KG. Then, we utilized the HKG representations for the DDI extraction task. Our proposed model incorporates HKG embeddings into the input sentence in the form of levitated markers and considers the relationship between contexts and KG information through an attention mechanism. In the experiment, we achieved an improvement of 1.70 pp on the DDIExtraction-2013 dataset by using HKG information. All types of nodes in the HKG contributed to the performance improvement of DDI extraction, and we showed the effectiveness of integrating heterogeneous information. We also evaluated the proposed method on the DrugProt dataset and achieved an F-score improvement of 1.4 pp using HKG information.

In future work, we would like to employ the deep neural entity linking model in our DDI extraction models. Jointly training HKG embeddings and BERT embeddings is also our future work. We also plan to apply our approach to other relation extraction tasks using the corresponding heterogeneous knowledge graphs.
